# The Predictive Value of Systemic Immune-Inflammation Index and Symptom Severity Score for Sepsis and Systemic Inflammatory Response Syndrome in Odontogenic Infections

**DOI:** 10.3390/jpm12122026

**Published:** 2022-12-07

**Authors:** Marius Pricop, Oana Ancusa, Serban Talpos, Horatiu Urechescu, Bogdan Andrei Bumbu

**Affiliations:** 1 Discipline of Oral and Maxillo-Facial Surgery, Faculty of Dental Medicine, “Victor Babes” University of Medicine and Pharmacy Timisoara, Eftimie Murgu Square 2, 300041 Timisoara, Romania; 2 Department V, Discipline of Medical Semiology I, Faculty of General Medicine, “Victor Babes” University of Medicine and Pharmacy Timisoara, Eftimie Murgu Square 2, 300041 Timisoara, Romania; 3 Department of Dental Medicine, Faculty of Medicine and Pharmacy, University of Oradea, 410073 Oradea, Romania

**Keywords:** systemic immune-inflammation index, disease severity score, odontogenic infections

## Abstract

Acute Odontogenic Infections (OI) are the leading cause of emergency visits and hospitalizations to the maxillofacial department, and may induce systemic inflammatory complications. Increasing numbers of OI patients need extended hospitalizations, various treatments, and intensive care. The Symptom Severity score (SS) helps doctors assess the likelihood of infection and admission complications. Systemic Immune-inflammation Index (SII) is a biomarker-based inflammatory prognosis score. It was hypothesized that greater SII and SS values might suggest a higher risk for sepsis and systemic inflammatory response syndrome (SIRS). Therefore, this research aims to discover whether SII and SS scores can reliably predict odontogenic infection severity and prognosis, and if they can be used to predict the development of SIRS and sepsis in OI using admission features. The study was designed as a retrospective cohort, with patients’ data being retrieved from medical records between January 2017 to April 2022. A total of 108 OI patients were matched 1:1 as low-severity and high-severity groups. Most individuals with severe infections had diabetes and smoking as comorbidities. Severe patients had longer hospital stays (12.0 days vs. 4.1 days), although mortality rates did not significantly differ. A total of 11.1% lower-severity patients (Group A) had SIRS during hospitalization, compared to Group B with 25.9%. Group A had 7.4% of patients that developed sepsis compared to Group B’s rate of 22.2%. The correlation between OI’s SS and SII index values was positive and statistically significant (r = 0.6314). The total SII index mean was 1303, whereas the mean values by severity were 696.3 in Group A and 2312.4 in Group B. Group A’s mean SS score was 6.1, while Group B’s was 13.6. According to the calculated AUC plots, SII and SS scores were accurate predictors of sepsis and SIRS development using OI admission parameters. The adjusted odds ratio for SIRS in OI patients was 2.09, and 2.27 for sepsis. Medical professionals and dentistry teams should be encouraged to use the SII and SS scores to diagnose and anticipate sepsis and SIRS, hence improving disease management decisions.

## 1. Introduction

Acute Odontogenic Infections (OI) are the main reason for emergency room visits and hospital admissions to the maxillofacial department [1]. These infections are often localized in the oral cavity, but, in their most severe forms, may progress to deeper spaces of the head and neck, resulting in airway obstruction, multiple organ failure, and even patient death [2]. More than that, in particular situations, odontogenic infections can possibly disseminate and cause systemic inflammatory complications. Estimating the severity of odontogenic infections is particularly important for predicting the disease’s prognosis and planning an effective therapy [3].

OI is often caused by decaying teeth and periodontal disease due to poor oral hygiene, leading to inflammation of pericoronal tissues [4]. In most cases, odontogenic infections are multimicrobial and caused by endogenous oral flora. Surgical incision, drainage, and tooth extraction, followed by antibiotic therapy, continue to be the cornerstones of treatment [5]. However, the management of OI has become increasingly complex, with an increasing number of patients needing lengthy hospitalizations, multiple interventions, and intensive care follow-ups [6]. Clinical signs such as dysphonia, dyspnea, restriction of tongue movement, and oropharyngeal edema alert odontologists and oral and maxillofacial surgeons to the severity of an infection. The increasing proportion of patients with underlying disorders such as alcoholism, immunodepression, or long-term diabetes [7] might explain the trend toward more severe infections, in addition to the increasing antibiotic resistance. A delay in diagnosis that is treated with many antibiotics or anti-inflammatory medications may not cure an illness, but rather alleviate its symptoms. As such, the Symptom Severity score (SS) was designed to help physicians correctly evaluate the risk of infection and possible complications that might occur during admission.

Inflammatory markers measured by blood tests are commonly used to predict the severity of odontogenic infection. One of the routine methods often used in clinical practice is to assess C-reactive protein (CRP), white blood cell count (WBC), and its fractions (neutrophils, lymphocytes, monocytes). However, their values alone have limited predictability to determine the severity of infection accurately, and an alternative examination is needed. An inflammatory prognostic score using patients’ biomarkers, the Systemic Immune-inflammation Index (SII), has recently been developed [8]. This index, based on peripheral platelet, neutrophil, and lymphocyte count, has been proven to be a promising prognostic indicator in various inflammatory diseases, including malignant tumors [9,10], coronary artery disease [11], acute ischemic stroke [12], and several chronic systemic diseases [13]. Its application in infectious diseases has not yet been fully defined, moreover, in the case of OI.

To the best of our knowledge, no studies have investigated the relationship between SII and SS and the severity of odontogenic infections. Therefore, it was hypothesized that higher Systemic Immune-inflammation Index (SII) values and a higher SS score might indicate an increased severity score in patients with odontogenic infections and higher odds of developing sepsis and systemic inflammatory response syndrome (SIRS). This study aimed to investigate whether the SII and SS scores might accurately predict the severity and prognosis of odontogenic infections and to determine their potential use in prediction models for SIRS and sepsis in OI.

## 2. Materials and Methods

### 2.1. Study Design and Patient Selection Process

The study was designed as a retrospective cohort of patients admitted for odontogenic infections to the Maxillofacial Surgery Department of City Emergency Hospital Timisoara (SCMUT), affiliated with the Victor Babes University of Medicine and Pharmacy from Timisoara between January 2017 to April 2022. These data were collected from digital and paper records only with the patient’s agreement and the ethical approval obtained from the Ethics Committee of SCMUT with the approval number I-27098 from 14 October 2022.

Adult patients aged eighteen and over were included in the study. The diagnoses considered for inclusion were infections of odontogenic origin, such as dentoalveolar abscess and cellulitis, according to the ICD-10 disease classification [14]. Patients with incomplete medical records were excluded from the study. Patients aged under 18 years, pregnant women, and patients with cancer, immunodeficiency, or infections of regions other than the oral cavity (non-odontogenic head and neck infections, posttraumatic infections) were not included in the study.

### 2.2. Patient Selection Process and Scale Assessment

The patient examination was performed according to the guidelines of the Maxillofacial Department of SCMUT. Dental X-rays were taken to assist with the diagnostic procedure. On admission, clinical status and vital signs were performed on all patients before the blood test. Antecubital venous blood was drawn on admission and on the day of hospital discharge. Body temperature was measured orally at least twice daily. All laboratory parameters were assessed by admission before antibiotic therapy. Entire patient management was comparable among all the patients. All patients were treated with similar surgical approaches and antibiotic therapy. Patients were discharged from the hospital after switching from intravenous to oral administration of antibiotics to complete the treatment plan when they were afebrile and clinically improved. The main clinical criteria for improvement were body temperature <38.3 °C, decreased edema or erythema, cessation of trismus and other specific symptoms on admission, and normalization of biological values indicative of infection.

Eligible cases were classified into two groups: the low-severity infection group, which included low to mild infections, and the increased-severity infection group, comprising moderate to severe infections, according to the Symptom Severity score (SS) of the symptoms assessed in this study [15] and presented in Table 1. The SS score of odontogenic infection developed by Sainuddin et al. [15], which was used in this research, was determined at admission. The score is based on several characteristics, including clinical manifestations of systemic inflammation known as Systemic Inflammatory Response Syndrome (SIRS) [16,17], which contains easily obtained laboratory results and clinical parameters that were readily and rapidly available to all clinicians [18]. Sepsis was defined by the recent guidelines in accordance with the sequential sepsis-related organ failure assessment score (SOFA) [19].

The severity of infection was also stratified by the compromised anatomic space, as follows: (1) Mild risk: canine, vestibular maxillary and mandibular, palatal involvement; (2) Moderate risk: submandibular, sublingual, submental, pterygoid-mandibular, sub-masseteric, or temporal; (3) Severe risk: Retropharyngeal, pterygoid-palatal, pre-tracheal, pterygoid-pharyngeal; (4) Extreme risk: mediastinum, intracranial, or prevertebral. Other factors that play a role in determining the degree of severity are the body’s immune system and concomitant systemic diseases such as diabetes mellitus, which may reduce body resistance to infections. Another parameter that plays a key role in determining the severity of infection is the assessment of one or more fascial spaces, as described in Table 1.

To determine the SII score, whole blood samples of 1.0 mL were routinely obtained from all patients at hospital admission, and a routine blood examination was performed immediately at admission. The SII was calculated from the platelet counts (reference range: 150–410 × 10³/μL), neutrophil counts (reference range: 2.04–7.60 × 10³/μL), and lymphocyte counts (reference range: 1.0–3.0 × 10³/μL), using the following formula: SII = platelet × neutrophil/lymphocyte counts [20]. The SII is expressed as × 10³/μL.

The sample of patients comprised 141 eligible cases diagnosed clinically and radiologically with odontogenic infections who were hospitalized in the Maxillofacial Surgery Department of the SCMUT between January 2017 to April 2022. Sample size was not calculated, due to the low number of patients with odontogenic infection, rather being constituted by including all available cases from the hospital database. After eliminating incomplete files and filtering by severity scores, a total of 108 patients were finally included in the analysis, being matched 1:1 by severity index. The records were further subcategorized according to main anatomic space involvement, and the SS score into two groups: Group A—the lower severity group (SS score from 0 to 8); Group B—the higher severity group (SS score from 9 to 16 points), each comprising 54 patients.

### 2.3. Data Collection and Variables

We collected demographic data and targeted data such as the site of infection, results of hematologic and biochemical parameters, markers of inflammation and infection on admission, pre-existing medical history, conditions associated with a potential for immunosuppression, diabetes mellitus status, chronic kidney disease (CKD), comorbidities that negatively impact current health status (obesity, smoking) and duration of stay. The hospital information system obtained the patients’ discharge reports, clinical evaluations, laboratory values, and imaging tests. Furthermore, routine blood samples, white blood cell count (WBC), hemogram indexes such as neutrophil and lymphocyte count, Neutrophil to Lymphocyte Ratio (NLR), Platelet count, and Systemic Immune-inflammation Index (SII) were evaluated. Mean values and standard deviations of the laboratory values were calculated.

The variables considered for analysis comprised demographic data: age, gender, and place of origin (urban, rural). Clinical presentation on hospital admission included: fever (body temperature > 37.5 °C), trismus (mild, moderate, or severe), odontalgia (visual analog scale), mandibular pain (visual analog scale), dysfunctional disturbances of the masticatory system (mandibular dysfunction, headache, and unilateral chewing side), edema, signs of obstruction (dyspnoea, dysphagia), and signs of systemic infection (temperature > 38.3 °C or <35.3 °C [21], heart rate (HR) >90 bpm, respiratory rate (RR) > 20/min, blood pressure (BP) and WBC < 4 or >12 × 10³/μL).

Routine blood sample on admission to the hospital: complete blood count (CBC), C-reactive protein (CRP mg/L), erythrocyte sedimentation rate (ESR mm/hour), blood glucose (g/dL), ionogram (sodium and potassium mmol/L), creatinine (mg/L) and the glomerular filtration rate was also calculated (GFR mL/min/1.73 m^2^), blood urea (mg/dL), transaminases: aspartate transaminase (AST IU/L), alanine transaminase (ALT IU/L), clotting time, swab culture with antibiogram. Research variables for serum parameters included the Neutrophil to Lymphocytes Ratio (NLR) values were obtained by dividing absolute Neutrophil and Lymphocytes counts. The SII was calculated as platelet count ×NLR; Systemic Inflammatory Response Syndrome (SIRS): containing clinical and paraclinical parameters, temperature, HR, RR, and WBC. The Symptom Severity score (SS) is based on scoring parameters such as SIRS, trismus condition, dysphagia, involvement of fascial space, the signs of dehydration, and comorbidities association.

### 2.4. Statistical Analysis

The Mann–Whitney U test was applied to compare non-normally distributed means, while Student’s *t*-test was used to compare normally distributed data. Kruskal–Wallis was used as a nonparametric approach that assesses for significant differences in a continuous dependent variable by a categorical independent variable (with two or more groups). Chi-square and Fischer’s exact tests were applied to verify a possible difference between the two groups regarding variables described as proportionate values. Regression analysis was applied to determine the association between SII and SS. We considered as statistically significant a correlation coefficient r with a value between 0.5023 to 0.7329. The hazard ratio and adjusted odds ratios were determined for the assessment of SII and SS scores as predictors for SIRS and sepsis. The area under the curve (AUC) was plotted for SII and SS to determine their accuracy in predicting SIRS and sepsis using admission features of patients with odontogenic infections. A *p*-value < 0.05 was considered statistically significant in comparison to the study variables.

The specific outcome measures included serum SII index (platelet count × NLR); each value was recorded in the study from the day of admission. Data were obtained electronically and de-identified. Mean values and standard deviations (SD), *p*-values, and correlation coefficient “r” of the laboratory values were calculated using the statistical analysis software MedCalc (MedCalc Software bv, Ostend, Belgium). Variables were compared between group A and group B, including the laboratory tests mentioned above related to the Severity Score (SS) of odontogenic infections.

## 3. Results

### Demographic Characteristics of the Study Population

In the current study, a total of 66 men (61.1%) and 42 women (38.9%) with a male-to-female ratio of 11:7 were evaluated. The mean age of group A was 39.5 years (age range 18–85), and the mean age of group B was 59.5 years (age range 20–81). Additionally, 57.4% of patients belong to the urban environment. Alongside age and gender distribution, Table 2 presents the environment of origin (rural and urban). Gender distribution presented no significant difference, but the age comparison showed a significant difference for the two groups (39.5 years in Group A vs. 59.5 years in Group B, *p*-value = 0.007). There was a significant difference between groups A and B regarding diabetes mellitus, which indicates a higher incidence among study group B according to *p*-value (51.9% vs. 18.5%, *p*-value < 0.001). In group A, there were nine (16.7%) patients who were current smokers, ten patients (18.5%) were diabetic, 31 (57.4%) were obese, 14 (25.9%) had chronic kidney disease, and five (9.3%) had a diagnosis of malignancy. In group B, 19 (35.2%) patients were current smokers, a significantly higher proportion compared to patients from Group A, 28 (51.9%) patients were diabetic, 37 (68.5%) were obese, 17 (31.5%) had chronic kidney disease, and seven (13.0%) had a diagnosis of cancer.

The main reason for hospitalization included cellulitis, abscess, periodontitis, and sepsis. The most common odontogenic cause was abscesses in 50.9% of the entire cohort, followed by abscesses associated with cellulitis in 37.9% of the studied patients. The most prevalent anatomical space infections were superficial lodges (44%), followed by peri-mandibular space (29.6%), while the least was fascial space (7.4%), as presented in Table 3. The proportion of patients with abscesses associated with cellulitis at admission was significantly higher in the higher severity group (Group B), with 55.6% patients, compared to 20.4% in Group A (*p*-value < 0.001). By location of the infection, there were no significant differences between the two groups with different severity of OI. The duration from symptom onset until hospital admission was significantly longer in patients with severe infection, with a median of 42.5 h in Group B, compared to 30.2 h in Group A (*p*-value < 0.001). A total of six (11.1%) patients in the lower severity group developed SIRS during hospitalization, a significantly lower proportion compared to those from Group B (25.9%, *p*-value = 0.047). Similarly, sepsis occurred during hospitalization in four (7.4%) patients from Group A, compared to 12 (22.2%) from Group B (*p*-value = 0.030). There were no ICU admissions among patients of lower severity odontogenic infection, but four patients from the higher severity group required intensive care (*p*-value = 0.041). As a consequence of higher severity, the median duration of hospitalization was significantly higher in Group B (12.0 days vs. 4.1 days, *p*-value < 0.001). However, the mortality rate did not differ significantly between the two study groups, as there were no cases of death in Group A and three cases (5.6%) in Group B.

Table 4 describes a comparison of severity scores among patients with odontogenic infections. A SIRS score of 3 or higher was calculated for seven patients in Group A (12.9%), compared to 70.4% among those in Group B (*p*-value < 0.001). Trismus score was normal in 55.6% of patients with lower severity OI, compared to 22.2% among those from group B (*p*-value < 0.001). The dysphagia score was moderate to severe in 17 (31.5%) patients from Group A, compared to 31 (57.4%) in Group B (*p*-value = 0.028). Lastly, fascial space scores and dehydration/comorbid scores were significantly increased in Group B patients with OI.

**Figure 1:**
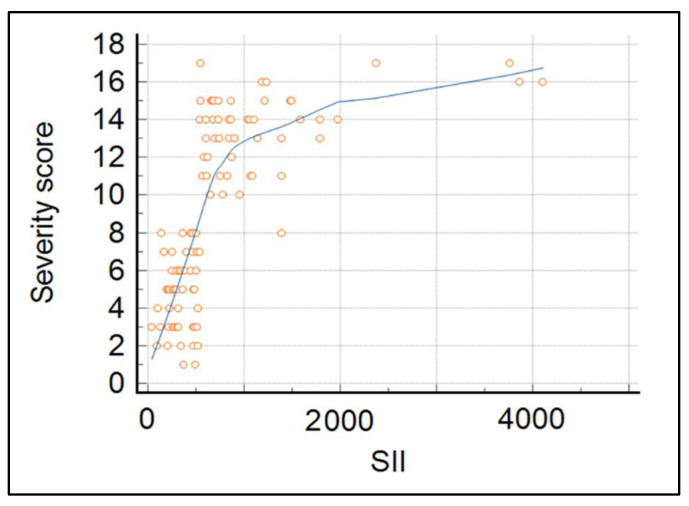
Scatter diagram of Symptom Severity Score and Systemic Immune-inflammation Index.

Correlation analysis was used to refine the initial results. Regression analysis of the correlation between the Severity Score (SS) of odontogenic infection with SII index values showed a strong correlation of r = 0.6314 with a *p*-value < 0.05. The significance level was set at 0.05. Therefore, there was a statistically significant correlation between the Symptom Severity score of odontogenic infections with SII index values (Figure 1). The overall SII index means value in the entire cohort was 1303, while the mean values by severity groups were 696.3 in Group A, compared to 2312.4 in Group B (*p*-value < 0.001). Similarly, there was a significant difference between SS scores, with a mean of 6.1 in Group A and 13.6 in Group B (*p*-value < 0.001).

**Figure 2:**
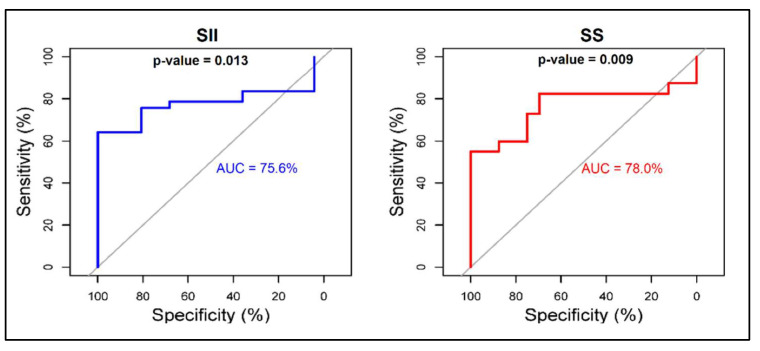
ROC plot for the accuracy of SS and SII in predicting sepsis using admission features in patients with odontogenic infections.

**Figure 3:**
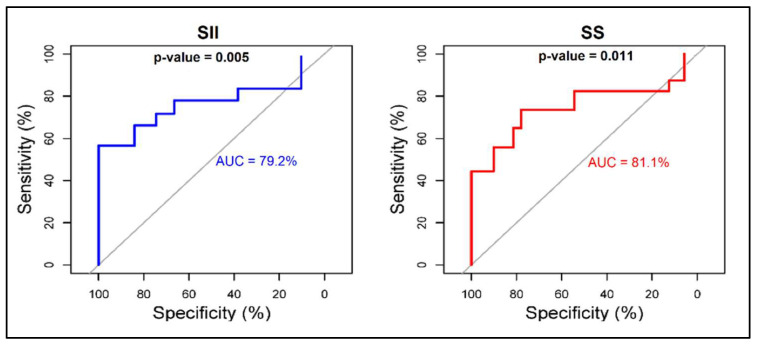
ROC plot for the accuracy of SS and SII in predicting SIRS using admission features in patients with odontogenic infections.

The hazard ratios (HR) and adjusted odds ratio (aOR) for SII and SS scores were calculated using admission parameters for predicting SIRS and sepsis after odontogenic infections. The adjusted odds ratio for the SII score regarding SIRS development in patients admitted with OI was 2.09 (95% CI = 1.16–5.17), while the SS score had a lower OR value for determining SIRS development, with an insignificant confidence interval (OR = 1.75, 95% CI = 0.98–2.83). When the risk was calculated for sepsis as the dependent variable, both SII and SS scores had a significant value after adjustment for age, comorbidities, and gender, as described in Table 5. The area under the curve (AUC) identified both the SII and SS scores as accurate predictors for the development of sepsis and SIRS using admission features of OI patients. As seen in Figure 2, the AUC for the SII score in predicting sepsis was 75.6%, compared to the AUC for the SS score of 78.0%. Lastly, the AUC for the SII score in predicting SIRS was 79.2%, compared to the AUC for the SS score of 81.1%, as seen in Figure 3.

## 4. Discussion

### 4.1. Supporting Literature

This study aimed to determine whether there is an association between elevated levels of inflammation serum markers calculated for the Systemic Immune-inflammation Index (SII) at admission and the Symptom Severity score (SS) in patients with odontogenic infections requiring hospital admission. Both scores were identified as predictors for sepsis and SIRS after OI, with good accuracy scores.

The Symptom Severity score of odontogenic infection is based on parameters that measure the inflammation state (SIRS, trismus, dysphagia, and fascial space collection) of the subject and factors (dehydration and comorbidities) that directly influence the subject systemic response to inflammation and odontogenic infections state [22,23]. In this study, patients were categorized into the less severe infection group (group A) if they had an SS < 9 points and into the more severe infection group (group B) if they had an SS > 9 points. An SS > 9 points were selected for the more severe group in this study because, by definition, high-risk space involvement is more likely to be present.

Inflammatory activity can be evaluated by a series of hematological indices derived from white blood cells: (WBC) and its elements, red cell distribution width, mean platelet volume, platelet, and combined ratios of these parameters such as Neutrophil-to-Lymphocyte Ratio (NLR), and Platelet-to-Lymphocyte Ratio (PLR) [24,25]. Hupp et al. stated that leukocytes respond early after infections, whereas there is a time delay in the production of CRP, peaking around 48 h after inflammation or initiation of infection [26]. Furthermore, Lippi et al. [27] show that blood-cell-count-derived inflammation indexes, including NLR and PLR, have been reported to be a more sensitive biomarker of inflammation than the individual levels of the blood cell line. Because their values exclusively have limited predictability to determine the severity of infection accurately, a prognostic indicator based on counts of neutrophils, lymphocytes, and platelets is expected to be more robust than one based on only a single factor [28].

In 2014, Hu et al. [29] developed an indicator called the Systemic Immune-inflammation Index, SII, to predict the prognosis of patients after curative resection for hepatocellular carcinoma. The SII was estimated from preoperative peripheral blood counts of platelets (P), neutrophils (N), and lymphocytes (L) per liter by the equation: SII = P × N/L. This index, based on peripheral platelet, neutrophil, and lymphocyte counts, has been proven to be a promising prognostic indicator in various inflammatory diseases, including malignant tumors, coronary artery disease, acute ischemic stroke, and several chronic systemic diseases [30]. Its application in infectious diseases has not yet been fully clarified. The utility of SII to identify patients at higher risk of developing severe infections is given by the differential roles that lymphocytes, neutrophils, and platelet play during the immune response. The lymphocytes are the only cells in the body capable of precisely recognizing and perceiving different antigens. They play a crucial role in most chronic inflammatory lesions, especially in autoimmune diseases and in diseases with persistent antigens. Neutrophils are the most important cellular defense against infections, and platelets contribute to hemostasis and participate in inflammation and host defense [31]. Considering these factors, SII might be better able to reflect the balance of host inflammatory and immune status. Therefore, H. Li et al. have shown that SII is a potential indicator of survival in COVID-19 [32].

Many of the existing studies related that CRP, white blood cell count (WBC), and its fractions (neutrophils, lymphocytes, monocytes) are often used as markers of inflammation and are reported to be almost useful in detecting maxillofacial infections [33,34]. In contrast, our study investigates the Systemic Immune-inflammation Index (SII) as a prognostic marker for the severity of odontogenic infections (OI). As a new type of inflammatory index, the SII is based on the absolute values of neutrophils, platelets, and lymphocytes in peripheral blood. It is easily obtained from routine blood tests, which confers an economic advantage to the method [35].

### 4.2. Study Limitations and Future Perspectives

There were several limitations to our study. First, our study was a single-center study of OI patients admitted to the hospital. Second, the number of patients was small. Another significant limitation of our study is its retrospective character which means that were dependent on medical records data, and in that way, statistical analysis is susceptible to human error. Within limits presented before, in our study, group B (the more severe infection group) had a significantly higher average of serum SII levels on admission than group A (the less severe infection group). However, there was a statistically significant difference between the mean serum SII index in the two groups. The higher the Severity Score is, the more proportional to the SII level. Extensive prospective studies should be performed to support our findings.

## 5. Conclusions

Infections of odontogenic etiology may result in sepsis and systemic inflammatory response syndrome, two potentially fatal complications resulting from an aberrant immune reaction to the infection. This may result in tissue damage, organ failure, and, ultimately, death. Recognizing and treating sepsis and SIRS promptly improves patient outcomes. Medical professionals and their dental teams should be taught to use the Systemic Inflammation Index (SII) and the Symptom Severity (SS) scores in the early diagnosis and prediction of sepsis and SIRS, hence possibly enhancing disease management decisions.

## Figures and Tables

**Table 1:** The Symptom Severity score (SS) of odontogenic infection developed by Sainuddin et al. [15].

Criteria		Score	Max Score
Systemic Inflammatory Response Syndrome (SIRS)	Temperature > 38.3 °C	1	4
	Heart rate > 90 bpm	1	
	RR 20/min	1	
	WBC < 4 or > 12 × 10^9^	1	
Trismus	Moderate < 2 cm	3	4
	Severe < 1 cm	4	
			
Dysphagia	Mild—able to swallow most foods	2	5
	Moderate—unable to swallow fluids	4	
	Severe—drooling saliva	5	
			
Collection in 1 fascial space	Low severity (canine, vestibular)	1	5
	Moderate severity (buccal)	2	
	High severity (all other spaces)	4	
Collection in 2 or more fascial spaces		5	
			
Sign of dehydration (↓BP/↑Urea/↓Skin turgor)		1	2
Comorbidities: diabetes mellitus, immunocompromised status, known or suspected chronic alcohol misuser		1	
			
Total Score			20

SIRS—systemic inflammatory response syndrome; BP—blood pressure; RR—respiratory rate; WBC—white blood cells.

**Table 2:** Comparison of background characteristics among patients with odontogenic infections.

Variables *	Lower Severity Group Group A (*n* = 54)	Higher Severity Group Group B (*n* = 54)	*p*-Value
Gender			0.236
Men	30 (55.6%)	36 (66.7%)	
Women	24 (44.4%)	18 (33.3%)	
Age, median (IQR)	39.5 (17.3)	59.5 (9.0)	0.007
Place of origin			0.436
Rural	21 (38.9%)	127 (46.3%)	
Urban	33 (61.1%)	127 (53.7%)	
Smoking			0.028
Yes	9 (16.7%)	19 (35.2%)	
No	45 (83.3%)	35 (64.8%)	
Comorbidities			
Diabetes mellitus	10 (18.5%)	28 (51.9%)	<0.001
Obesity	31 (57.4%)	37 (68.5%)	0.231
Chronic kidney disease	14 (25.9%)	17 (31.5%)	0.523
Malignancy	5 (9.3%)	7 (13.0%)	0.540
Others	2 (3.7%)	4 (7.4%)	0.401

* Data reported as n (%) and calculated using the Chi-square test and Fisher’s exact test unless specified differently; median and IQR values compared with Mann–Whitney U-test; IQR—Interquartile range.

**Table 3:** Comparison of infection characteristics among patients with odontogenic infections.

Variables *	Lower Severity Group Group A (*n* = 54)	Higher Severity Group Group B (*n* = 54)	*p*-Value
Reason for hospitalization			<0.001
Abscess	38 (70.4%)	17 (31.5%)	
Cellulitis	5 (9.3%)	7 (13.0%)	
Association of abscess and cellulitis	11 (20.4%)	30 (55.6%)	
Infection site			
Peri-maxillary	13 (24.1%)	10 (18.5%)	0.480
Peri-mandibular	14 (25.9%)	18 (33.3%)	0.399
Superficial lodges	22 (40.7%)	26 (48.1%)	0.438
Deep lodges	1 (1.9%)	2 (3.7%)	0.558
Fascial	5 (9.3%)	3 (5.6%)	0.462
Duration from symptom onset until hospital admission (hours), median (IQR)	30.2 (19.5)	42.5 (23.2)	<0.001
SIRS	6 (11.1%)	14 (25.9%)	0.047
Sepsis	4 (7.4%)	12 (22.2%)	0.030
ICU admission	0 (0.0%)	4 (7.4%)	0.041
Duration of hospitalization, median (IQR)	4.1 (2.8)	12.0 (5.7)	<0.001
Severe complications	2 (3.7%)	9 (16.7%)	0.025
Mortality	0 (0.0%)	3 (5.6%)	0.078

* Data reported as n (%) and calculated using the Chi-square test and Fisher’s exact test unless specified differently; median and IQR values compared with Mann–Whitney U-test; IQR—Interquartile range; ICU—Intensive care unit.

**Table 4:** Comparison of severity scores among patients with odontogenic infections.

Variables *	Lower Severity Group Group A (*n* = 54)	Higher Severity Group Group B (*n* = 54)	*p*-Value
SIRS score			<0.001
0	14 (25.9%)	5 (9.2%)	
1	18 (33.3%)	8 (14.8%)	
2	10 (18.5%)	8 (14.8%)	
3	6 (11.1%)	19 (35.2%)	
4	1 (1.8%)	19 (35.2%)	
Trismus score			<0.001
Normal	30 (55.6%)	12 (22.2%)	
Moderate	19 (35.2%)	15 (27.8%)	
Severe	5 (9.3%)	27 (50.0%)	
Dysphagia score			0.028
Normal	5 (9.3%)	18 (33.3%)	
Mild	21 (38.9%)	16 (29.6%)	
Moderate	17 (31.5%)	29 (53.7%)	
Severe	0 (0.0%)	2 (3.7%)	
Fascial space score			<0.001
Low risk	39 (0.0%)	10 (18.5%)	
Moderate risk	23 (42.6%)	27 (50.0%)	
Severe risk	0 (0.0%)	8 (14.8%)	
Dehydration/Comorbid			0.001
No dehydration and comorbid	28 (51.9%)	13 (24.1%)	
Dehydration or comorbid	26 (48.1%)	22 (40.7%)	
Dehydration and comorbid	3 (5.6%)	16 (29.6%)	
Severity scores, (mean ± SD)			
SS	6.1 ± 1.8	13.6 ± 3.9	<0.001
SII	696.3 ± 35.2	2312.4 ± 66.0	<0.001

* Data reported as n (%) and calculated using the Chi-square test and Fisher’s exact test unless specified differently; SD—standard deviation; SS—Severity Score; SII—Systemic Immune-inflammation Index.

**Table 5:** Hazard ratios and adjusted odds ratios for SII and SS scores were calculated at admission for predicting SIRS and sepsis after odontogenic infections.

Variables	Risk (95% CI)	*p*-Value
SIRS (dependent)		
Hazard ratio		
SII	3.25 (1.13–7.44)	<0.001
SS	2.39 (1.07–6.60)	<0.001
Adjusted odds ratio *		
SII	2.09 (1.16–5.17)	0.003
SS	1.75 (0.98–2.83)	0.044
Sepsis (dependent)		
Hazard ratio		
SII	3.84 (1.61–8.53)	<0.001
SS	3.20 (1.54–6.97)	<0.001
Adjusted odds ratio *		
SII	2.27 (1.30–5.42)	0.001
SS	2.04 (1.06–4.19)	0.022

* Data were adjusted for age, comorbidities, and gender; SD—standard deviation; SS—Severity Score; SII—Systemic Immune-inflammation Index; CI—Confidence Interval.

## Data Availability

Data available on request.
